# Exposure to a Slightly Sweet Lipid-Based Nutrient Supplement During Early Life Does Not Increase the Preference for or Consumption of Sweet Foods and Beverages by 4–6-y-Old Ghanaian Preschool Children: Follow-up of a Randomized Controlled Trial

**DOI:** 10.1093/jn/nxy293

**Published:** 2019-02-15

**Authors:** Harriet Okronipa, Mary Arimond, Rebecca R Young, Charles D Arnold, Seth Adu-Afarwuah, Solace M Tamakloe, Helena J Bentil, Maku E Ocansey, Sika M Kumordzie, Brietta M Oaks, Kathryn G Dewey

**Affiliations:** 1 Program in International and Community Nutrition, Department of Nutrition, University of California, Davis, CA; 2 *Intake* - Center for Dietary Assessment, FHI 360, Washington, DC; 3 Department of Nutrition and Food Science, University of Ghana, Legon, Ghana; 4 Department of Nutrition and Food Sciences, University of Rhode Island, Kingston, RI

**Keywords:** sweet food, sugar-sweetened beverage, preference, consumption, lipid-based nutrient supplements, children, Ghana

## Abstract

**Background:**

Whether consuming sweet foods early in life affects sweet food preferences and consumption later in childhood is unknown.

**Objective:**

We tested the hypothesis that exposure to a slightly sweet lipid-based nutrient supplement (LNS) early in life would not increase preference for or consumption of sweet items at preschool age.

**Methods:**

We followed up children who had participated in a randomized trial in Ghana in which LNS was provided to 1 group of women during pregnancy and 6 mo postpartum and to their infants from ages 6–18 mo (LNS group). The control group (non-LNS group) received iron and folic acid during pregnancy or multiple micronutrients during pregnancy and 6 mo postpartum, with no infant supplementation. At 4–6 y, we obtained data from caregivers on children's food and beverage preferences and consumption (*n* = 985). For a randomly selected subsample (*n* = 624), we assessed preference for sweet items using a photo game (range in potential scores, 0–15). For the photo game and reported consumption of sweet items, we examined group differences using predetermined noninferiority margins equivalent to an effect size of 0.2.

**Results:**

Median (quartile 1, quartile 3) reported consumption of sweet items (times in previous week) was 14 (8, 23) in the LNS group and 16 (9, 22) in the non-LNS group; in the photo game, the number of sweet items selected was 15 (11, 15) and 15 (11, 15), respectively. The upper level of the 95% CI of the mean difference between LNS and non-LNS groups did not exceed the noninferiority margins for these outcomes. Caregiver-reported preferences for sweet items also did not differ between groups (*P* = 0.9).

**Conclusion:**

In this setting, where child consumption of sweet foods was common, exposure to a slightly sweet LNS early in life did not increase preference for or consumption of sweet foods and beverages at preschool age. This trial was registered at clinicaltrials.gov as NCT00970866.

## Introduction

The prevalence of childhood overweight and obesity worldwide remains high (1, 2). This is a cause for concern because childhood overweight is a risk factor for adult obesity and its consequences (3). Among African children aged <5 y, the prevalence of overweight was 7% in 2011 and is expected to reach 11% by 2025 (4). One risk factor for childhood overweight is the consumption of foods high in added sugar. Several systematic reviews and meta-analyses have reported positive associations between the consumption of sugar-sweetened beverages (SSB) and weight gain, diabetes, and other chronic diseases (5–7). The WHO consequently recommends consuming no more than 5–10% of total energy intake from added sugars (8). Globally, intake of added sugar remains above the recommended levels (9–12).

Experimental research has shown that children's liking for sweet taste is inborn (13). However, it has been suggested that early dietary experiences may also play an important role in the development of preferences and the consumption of foods later in childhood (14, 15). Longitudinal studies in the United States have suggested that babies who were repeatedly or routinely fed sweetened water during the early months of life showed greater preference for sweetened water at ages 6 mo (16), 2 y (17), and 6–10 y (18). However, parental feeding practices relating to the feeding of sweet items during infancy may confound these associations (19). A recent systematic review on the subject concluded that the evidence regarding effects of exposure to sweet tasting foods and beverages on subsequent generalized acceptance or preference for sweet tasting foods and beverages was equivocal (20).

In recent years, home fortification interventions including the use of small-quantity lipid-based nutrient supplements (LNS) have been evaluated as part of strategies to address undernutrition during the first 1000 days of life. The supplements are made from peanuts, milk powder, vegetable oil, and multiple micronutrients (MMN). We refer to the LNS used in this study as “slightly sweet” because they contained a small amount of added sugar (∼1.6 g/20 g LNS). Acceptability studies in different settings showed that they are widely acceptable (21–23). Studies have shown these supplements to have the potential to improve child growth and development in some contexts (24–28). It is unlikely that consumption of this small amount of sugar in LNS would impact the preference for or consumption of sweet foods and beverages later in childhood. However, as LNS are a novel product, any potential impacts on later preference for or consumption of sweet items need to be examined.

The present study aimed at examining the long-term impact of early exposure to LNS on the preference for or consumption of sweet foods and beverages later in childhood. To rule out any potential adverse effects, we tested the hypothesis that the preference for or consumption of sweet foods and beverages by children who were exposed to LNS early in life would not be higher than those of children who were never exposed to LNS, using a noninferiority approach. We also examined whether the consumption of peanut-containing foods as well as other foods differed between the LNS and the non-LNS groups.

## Methods

### Study design and participants

The study reported here was a follow-up study of children whose mothers participated in the iLiNS-DYAD Ghana trial.

#### The iLiNS intervention trial (2009–2014)

The iLiNS-DYAD Ghana supplementation trial was conducted in the Manya Krobo and Yilo Krobo districts in the Eastern Region of Ghana between December 2009 and March 2014. The area is semi-urban and is located about 70 km northeast of the national capital Accra, with study communities extending along a 20 km stretch of the main road.

Details of the study design, randomization, and recruitment have been published elsewhere (25, 29). Briefly, the iLiNS-DYAD trial was a partially double-blind, individually randomized controlled trial that enrolled 1320 pregnant women aged ≥18 y at ≤20 weeks of gestation attending antenatal clinics in the 4 main health facilities in the study area. The women were randomly assigned to 1 of 3 supplements daily: (a) iron and folic acid capsule during pregnancy and a calcium placebo during the first 6 mo postpartum with no infant supplementation (IFA group), (b) MMN capsule [18 vitamins and minerals provided at levels of 1–2 times the recommended nutrient intakes (30)] during pregnancy and the first 6 mo postpartum, with no infant supplementation (MMN group), or (c) 20 g/d (118 kcal/d) lipid-based nutrient supplement (LNS) during pregnancy and the first 6 mo postpartum followed by infant LNS supplementation (20 g/d) from ages 6–18 mo (LNS group). The LNS had the same micronutrient content as the MMN supplement, plus calcium, magnesium, phosphorus, potassium, and macronutrients (essential fatty acids and a small amount of protein). Women were in the trial from pregnancy to 6 mo postpartum and the children born to them from birth until age 18 mo.

#### The iLiNS follow-up study (2016)

**FIGURE 1:**
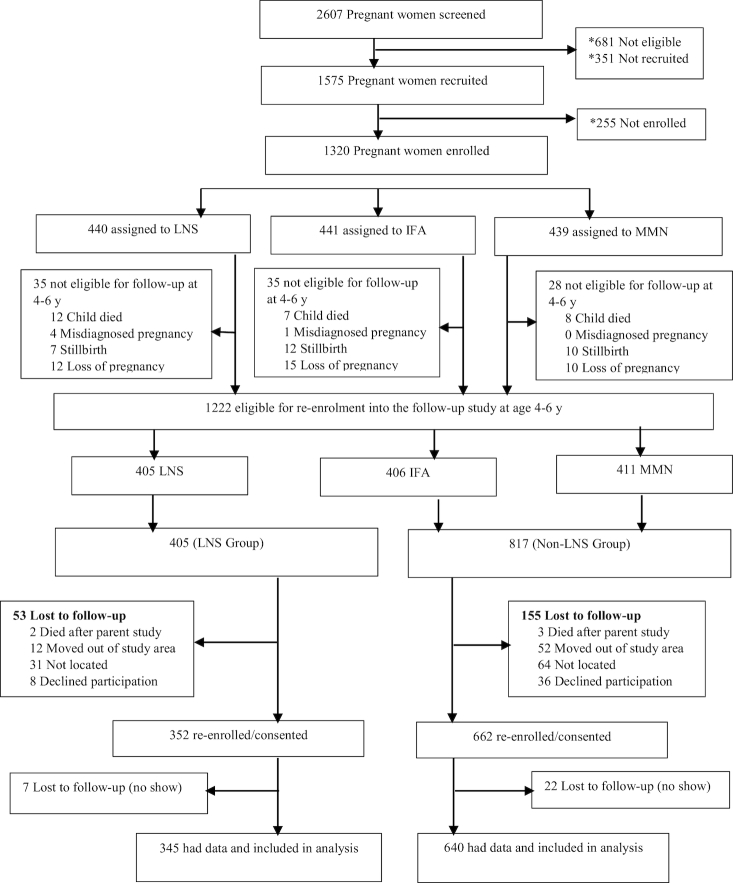
Study profile. IFA, iron and folic acid; LNS, lipid-based nutrient supplement; MMN, multiple micronutrients. LNS group, women received 20 g LNS daily during pregnancy and for 6 mo postpartum. Infants received 20 g LNS daily from 6–18 mo of age; Non-LNS group, women received either IFA during pregnancy and placebo for 6 mo postpartum or MMN capsules during pregnancy and for 6 mo postpartum. Infants did not receive any supplement. *Details reported in (29).

Between January and December 2016, we conducted a follow-up study (iLiNS-DYAD-G2) of children who participated in the iLiNS-DYAD Ghana intervention trial (herein referred to as “parent trial”) to examine the long-term impact of the intervention on the preference for or consumption of sweet foods and beverages and other outcomes. At the time of the follow-up study, the children were aged 4–6 y. Before the start of data collection for the follow-up study, we re-established contact with participants to update their contact information. This made it easy to locate them for re-enrolment into the follow-up study. All children enrolled in the parent trial, who were alive at the time of the follow-up, were eligible (**Figure 1**, *n* = 1222) to be invited to be a part of the follow-up study, irrespective of whether they remained in the trial at endline (age 18 mo).

### Data collection procedures

Before the follow-up study, we carried out a pilot study on 30 preschool children between the ages of 4 and 6 y to examine the feasibility and cultural acceptability of the methodology proposed for measuring sweet taste and sweet food and beverage preference in the follow-up study. Results from the pilot study showed that the proposed methodology was feasible and culturally acceptable.

Before the start of data collection for the follow-up study, we first contacted mothers or caregivers to inform them of the study. If they were interested, study personnel provided more details of the study procedures and obtained informed consent after which sociodemographic information was collected. A second home visit was scheduled to obtain information from the mother/primary caregiver (herein referred to as caregiver) on the food and beverage preferences and consumption of the child using a questionnaire. After this information had been collected, the caregiver was then invited to bring the child to the study test center for other testing (photo game, described below). Transportation costs incurred on the day of testing were reimbursed. The study protocols were approved by the institutional review boards of the University of California, Davis, the Ethics Committee for the College of Basic and Applied Sciences at the University of Ghana, and the Ghana Health Service Ethical Review Committee. The follow-up study was part of the iLiNS-DYAD Ghana trial, which was registered at clinicaltrials.gov as NTC00970866. Caregivers and study personnel were not informed of the study hypothesis and study personnel were blinded to group assignment of the children.

#### Caregiver report of child's food and beverage preferences and consumption

To obtain information regarding the child's food and beverage preferences and consumption as reported by the caregiver, we adapted the approach used by Skinner et al. (31) and Park et al. (32). Each caregiver completed a 2-part food and beverage preference and consumption questionnaire during an in-home interview. The first part of the questionnaire asked about the number of times different food and beverage items (or food/beverage groups) were consumed by the child in the week preceding the interview. The different food items were grouped into the following: SSB; sweet foods eaten as snacks or with meals; savory foods eaten as snacks or with meals; peanut-containing foods; foods containing eggs, fish, or meat; vegetables and fruits. Specific examples of these foods were given on the questionnaire and were informed by our knowledge of the local diet as well as from information available from the parent trial and from information gathered from a pilot study (mentioned above) before the follow-up study.

The second part of the questionnaire assessing food preferences consisted of a checklist of 30 specific food and beverage items commonly consumed by children aged 4–6 y in the study area. The list was developed based on key informant interviews with women in the study area in the pilot study mentioned above. The checklist included: 5 sweet low nutrient-dense beverages; 5 fruits; 5 sweet low fat, low nutrient-dense foods; 5 sweet, high fat, low nutrient-dense foods; 5 vegetables; and 5 savory, high fat, low nutrient-dense items (**Supplemental Table 1**). For each food and beverage item, primary caregivers were asked to indicate the child's preference by reporting whether the child liked the food item very much, liked it a little, disliked it, had never been offered or never tried the item, or the caregiver did not know. The questionnaire was administered at home during scheduled home visits.

#### Food and beverage preference as indicated by child (photo game)

We measured the food and beverage preferences of the child using an innovative photographic game method. We adapted the approach used by Boyland et al. in their Leeds Food Preference Measure, which involved a written checklist of 32 items and required that the participant mark next to an item if he/she would like to eat it at that moment (33). For our study, because the children were younger than the children in the Boyland study, and because we were concerned that their reading skills may not have been sufficient for a written checklist, we used pictures instead and used a game approach.

The food items used in the photo game were the same 30 specific food and beverage items that were included in the checklist for the second part of the caregiver questionnaire described above (Supplemental Table 1). We photographed the food and beverage items (displayed on a white background) as sold (or served) and consumed [the serving size and packaging most commonly encountered in local shops (for items purchased)] to ensure familiarity.

We conducted the photo game in a closed room at our testing center (located at a central location in the study area). A trained research assistant conducted the test. The test room was partitioned into 2 sections, 1 to familiarize the children with testing procedures using different children's toys and 1 to conduct the actual photo game, which had 2 levels. In the first level, children were requested to choose as many items as they would like to have “right now” (assuming the item was available) out of a total of 30 food and beverage items (15 of which were sweet). In the second level of the game, children were requested to choose their favorite 5 food and beverage items out of the total items they selected in the first level. Questions were also included on the questionnaire to assess whether the child knew or recognized each of the 30 food and beverage items. This assessment was done before the child made any selection. Familiarity with each item was classified as follows: knows the name of food or beverage item and has consumed it; knows the name of food or beverage item but has not consumed it; child gave the wrong name, but after hearing correct name, says he/she has consumed it; child could not name item, but after hearing correct name, says he/she has consumed it. The research assistant recorded the child's food and beverage choices on a tracking grid.

### Outcome variables

For the caregiver report of child consumption of sweet foods and beverages, we first summed up the number of times per week the child consumed all the different sweet foods and beverages listed on the questionnaire after which we calculated the number of times per week the child consumed sweet foods and beverages. A similar approach was used to calculate the number of times per week the child consumed SSB, peanut-containing foods, and other food groups.

For the report of child's food and beverage preferences based on interviews with the caregiver, we calculated a preference score by first assigning individual scores to caregiver responses to the question on “how much the child liked specific food/beverage items,” as follows: likes very much, 2; likes a little, 1; dislikes, −1; never offered/tried or don't know, 0. The scores were then summed to obtain a total preference score for each 5-item food category, ranging from −5 to 10. Higher scores indicated higher preference.

For each level of the photo game, we defined sweet food and SSB preference as the total number of photos of sweet items (values range from 0 to 15) chosen by the child out of 30 food and beverage items.

### Sample size calculations and statistical analysis

Our main outcomes were (a) the number of photos of sweet food/beverage items chosen by the child in the first level of the photo game, (b) the number of times per week the child consumed sweet foods and beverages as reported by caregiver, and (c) the number of times per week the child consumed SSB as reported by caregiver. For these outcomes, differences between the LNS and non-LNS (combined IFA and MMN) groups were compared using a noninferiority approach. The sample size was calculated based on detecting a small effect size, Cohen's *d*, of 0.2 (34), equivalent to a noninferiority margin of 0.66, 1.96, and 1.08 items, respectively, for these 3 outcomes based on SD of 3.3, 9.8, and 5.4 items, respectively (obtained from pilot data as well as preliminary data from the study). This yielded a minimum sample size of 620 (310 per group) assuming 80% power and α 0.05%. Accounting for 25% attrition increased the total minimum sample size to 775 (388 per group). Given that 1222 children were eligible for follow-up, we aimed to recruit a subsample of 775 for the photo game, randomly selected from all eligible children, and to collect data on all of the children we could locate for the other outcomes.

A statistical analysis plan was posted on our website (www.ilins.org) before data analysis. We examined whether children in the 2 groups differed in maternal, child, and household characteristics, using ANOVA for continuous variables and chi-square tests for categorical (binary) variables. We also examined differences in these characteristics between children for whom data were available and those who were lost to follow-up. We examined differences in outcomes between treatment groups with the use of negative binomial and ANCOVA modeling techniques. Because results from these 2 modelling techniques were similar, we present the results from the ANCOVA models for ease of interpretation. We compared groups using 2 models. The first model adjusted for child age at follow-up only. The second model also adjusted for the following prespecified covariates if they were significantly associated with the outcome at *P* < 0.10 in bivariate analysis: child sex; maternal education, prepregnancy BMI, age and nulliparity at baseline (time of enrolment into the parent trial); household asset score and distance of participant's house from the weekly market at baseline. We also examined group differences in the number of times per week the child consumed peanut-containing foods and other foods as well as in the preference score for sweet food/beverage items as reported by the caregiver.

All hypotheses tests except the noninferiority hypotheses were 2-sided at a 5% level of significance. For the noninferiority hypotheses, mean differences between groups in food and beverage preferences and consumption were compared with prespecified noninferiority margins (listed above). Noninferiority was deemed established if the difference in the mean and the 95% CI around it fell below the noninferiority margin (where lower is better). For all figures showing the noninferiority results, we re-scaled all outcome values to SD units for clarity.

For the photo game, we conducted a sensitivity analysis to include a chosen food and beverage item only if the child knew the name, irrespective of whether or not they had ever consumed it. In addition, we conducted a sensitivity analysis in which we included all 5 fruit items in the sweet food and beverage category. We examined the association between preference variables based on the photo game and those based on caregiver report using Spearman's correlation analysis. All analyses were carried out using SAS for Windows Version 9.4 (SAS Institute). Data analysts were fully blinded to group assignments until analyses were completed.

## Results

### Participants at follow-up

We successfully re-enrolled 83.0% (*n* = 1014) of all eligible children (*n* = 1222) into the follow-up study (Figure 1). We obtained data from caregivers on child food and beverage preference and consumption for 985 children (345, LNS; 640, non-LNS), which constituted 74.6% of all women who were enrolled into the parent trial and 80.6% of participants who were eligible for follow-up. The proportion of children lost to follow-up was significantly higher in the non-LNS group than in the LNS group (27.3% compared with 21.6%, *P* = 0.025). There were no differences in baseline characteristics between children included in the analysis and children lost to follow-up except that mothers of children included in the analysis were less likely to be nulliparous (*P* = 0.017) (**Supplemental Table 2**).

**Table 1** compares maternal, household, and child characteristics between the 2 intervention groups. At the time of enrolment into the parent trial, on average, women were aged ∼26 y and had about 8 y of formal education, and most (>90%) were married or cohabiting with a partner. At follow-up, children were 4.9 ± 0.6 y old, weighed 16.6 ± 2.2 kg, had a mean height of 106.4 ± 5.5 cm, and a mean BMI-for-age z score of −0.6 ± 0.8 SD. Approximately 3% of children were overweight (BMI-for-age zscore > +1 SD) at follow-up. We found no differences in most characteristics between the 2 groups except that children in the LNS group were from households with lower mean asset scores compared with the non-LNS group (−0.07 ± 0.96 compared with 0.07 ± 0.96, *P* = 0.031).

**TABLE 1:** Selected maternal and child characteristics, by intervention group for participants who had data on child food/beverage preference or consumption (as reported by caregiver)^1^

Variable	All groups combined (*n* = 985)	LNS group (*n* = 345)	Non-LNS group (*n* = 640)	*P* value^2^
Maternal characteristics at baseline				
Age, y	26.8 ± 5.4	26.9 ± 5.5	26.8 ± 5.4	0.7
Education, y	7.6 ± 3.5	7.7 ± 3.8	7.6 ± 3.4	0.9
Married or cohabiting, *n* (%)	919 (93.3)	319 (92.5)	600 (93.7)	0.4
Prepregnancy BMI, kg/m^2^	24.6 ± 4.4	24.9 ± 4.4	24.4 ± 4.5	0.1
Nulliparity, *n* (%)	315 (32.0)	110 (31.8)	205 (32.0)	0.9
Household assets index^3^	0.03 ± 0.96	−0.07 ± 0.96	0.07 ± 0.96	0.031
Distance to market, m	1239 (655, 2327)	1313 (703, 2387)	1223 (617, 2254)	0.2
Child characteristics at follow-up				
Male sex, *n* (%)	474 (48.2)	166 (48.1)	308 (48.2)	0.9
Age, y	4.9 ± 0.6	4.9 ± 0.6	4.9 ± 0.6	0.1
Height, cm	106.4 ± 5.5	106.9 ± 5.8	106.1 ± 5.4	0.056
Weight, kg	16.6 ± 2.2	16.7 ± 2.2	16.5 ± 2.2	0.056
BMI-for-age z score	−0.6 ± 0.8	−0.5 ± 0.8	−0.6 ± 0.8	0.5
Overweight,^4^*n* (%)	28 (2.8)	10 (2.9)	18 (2.8)	0.9

^1^Values represent mean ± SD or *n* (%) or median (quartile 1, quartile 3); LNS, lipid-based nutrient supplement; non-LNS, no exposure to lipid-based nutrient supplement (control group).

^2^Group differences were compared using ANOVA for continuous variables and the chi-square test for categorical variables.

^3^Proxy indicator for socioeconomic status constructed for each household based on ownership of a set of assets (radio, television, refrigerator, cell phone, and stove), lighting source, drinking water supply, sanitation facilities, and flooring materials. Household ownership of these sets of assets was combined into an index (with a mean of 0 and SD of 1) using principal components analysis. A higher value represents a higher socioeconomic status.

^4^Overweight defined as BMI-for-age zscore > +1 SD.

### Children's preference for sweet foods and beverages

Of the 775 children randomly selected for the photo game, 123 could not be re-enrolled for the following reasons: not located (*n* = 58); moved out of study area (*n* = 41); child died before follow-up study (*n* = 3); and declined participation (*n* = 21). Of the 652 who were re-enrolled, 624 successfully completed the photo game: 301 from the LNS group and 323 from the non-LNS group. Twenty-eight children (LNS = 12, non-LNS = 16) did not complete the test because they did not attend (*n* = 25), refused testing (*n* = 1), or could not focus on the game (*n* = 2). There were no differences in most child and household characteristics of those with and without photo game data (**Supplemental Table 3**). However, at baseline mothers of children who did not participate in the photo game were younger, less likely to be married, more likely to be nulliparous, and had lower prepregnancy BMI than those whose children had participated.

**FIGURE 2:**
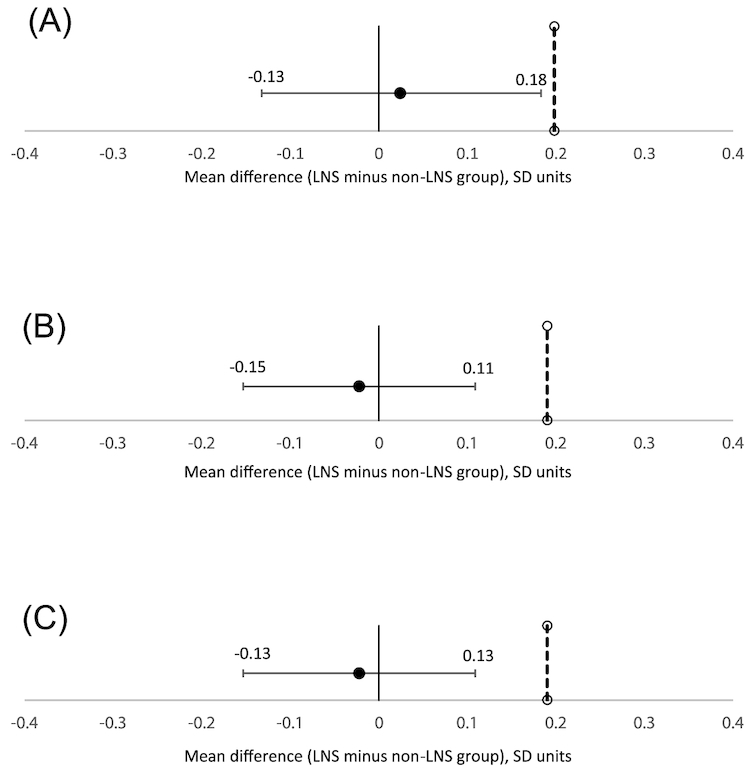
Noninferiority graphs. All outcome values have been re-scaled to SD units. Error bars indicate 95% CIs. The noninferiority margin is denoted by the dotted line. (A) Difference in children's preference for sweet foods and beverages (defined as the number of sweet items chosen by child from among 30 food and beverage items included in a photo game) between the LNS (*n* = 301) and non-LNS groups (*n* = 323). The 95% CIs lie to the left of the noninferiority margin (0.2 SD), indicating noninferiority (i.e., the preference for sweet foods and beverages by the LNS group was not higher than that preferred by the non-LNS group). Analysis adjusted for household assets and distance to weekly market. (B) Difference in children's consumption of sweet foods and sugar-sweetened beverages (as reported by caregiver) between LNS (*n* = 345) and non-LNS groups (*n* = 640). The 95% CIs lie to the left of the noninferiority margin (0.2 SD), indicating noninferiority (i.e., the preference for sweet foods and beverages by the LNS group was not higher than that preferred by the non-LNS group). Analysis adjusted for prepregnancy BMI, maternal age, maternal education, nulliparity, and household assets. (C) Difference in children's consumption of sugar-sweetened beverages (as reported by caregiver) between LNS (*n* = 345) and non-LNS groups (*n* = 640). The 95% CIs lie to the left of the noninferiority margin (0.2 SD), indicating noninferiority (i.e., the consumption of sweet foods and beverages by the LNS group was not higher than that consumed by the non-LNS group). Adjusted for prepregnancy BMI, maternal education, nulliparity, and household assets. LNS, lipid-based nutrient supplement; non-LNS, no exposure to lipid-based nutrient supplement (control group).

On average, children spent 20.6 ± 3.0 min completing the photo game. **Table 2** shows the total number of sweet items selected out of 30 food and beverage items in the first and second levels of the game. The mean difference (95% CI) between the LNS and the non-LNS groups in the number of sweet items chosen during the first level was 0.06 (−0.47, 0.58). The upper level of the 95% CI of this mean difference between the 2 groups (unadjusted or adjusted for covariates) did not cross the noninferiority margin (**Figure 2**A), indicating that the LNS group did not have a higher preference for these items compared to the non-LNS group. When children selected their top 5 food items, the number of sweet items selected was similar between the 2 groups (Table 2).

**TABLE 2:** Sweet food and beverage preference among 4–6-y-old Ghanaian children who participated in the iLiNS-DYAD-G2 follow-up study, by intervention group^1^

			Adjusted for child age at follow-up		Adjusted for baseline and other covariates	
			LNS vs. No LNS		LNS vs. No LNS	
Variable	LNS group Median (Q1, Q3)	Non-LNS group Median (Q1, Q3)	Difference in means (95% CI)	*P* value	Difference in means (95% CI)	*P* value
Preference as assessed in photo game (*n* = 624)	*n* = 301	*n* = 323	—		—	
All food/beverage items						
Total items chosen out of 30 items	28 (19, 30)	28 (21, 30)	—		—	
Number of sweet items chosen by child from among 30 items^2^	15 (11, 15)	15 (11, 15)	0.06 (−0.47, 0.58)	0.8	0.08 (−0.44, 0.61)^3^	0.7
Number of sweet items chosen by child (out of top 5 favorite food/beverage items)	3 (3, 4)	3 (2, 4)	0.09 (−0.08, 0.27)	0.3	0.07 (−0.10, 0.24)^4^	0.4
Only food/beverage items that were known^5^						
Total items chosen	14 (10, 18)	14 (10, 17)	—		—	
Number of sweet items chosen by child from among 30 items^2^	8 (5, 10)	8 (5, 9)	0.13 (−0.31, 0.58)	0.5	0.22 (−0.21, 0.66)^6^	0.3
Number of sweet items chosen by child (out of top 5 favorite food/beverage items)	2 (1, 3)	2 (1, 3)	0.05 (−0.13, 0.24)	0.6	0.06 (−0.12, 0.25)^7^	0.5
Preference as reported by caregiver (*n* = 985)	*n* = 345	*n* = 640	—		—	
Preference score for sweet food/beverage items (as reported by caregiver)^8^	25 (21, 28)	25 (21, 28)	0.14 (−0.60, 0.88)	0.7	−0.05 (−0.78, 0.69)^9^	0.9

^1^Group differences compared using multiple linear regression and ANCOVA models. LNS, lipid-based nutrient supplement; non-LNS, no exposure to lipid-based nutrient supplement (control group); Q, quartile.

^2^Group differences were compared using a noninferiority margin of 0.66. Noninferiority testing should take precedence over the *P* value (see Figure 2A for graph).

^3^Adjusted for household assets and distance to weekly market.

^4^Adjusted for child age only.

^5^This subanalysis only included food/beverage items that were known and recognized by the child (based on specific questions described in methods above).

^6^Adjusted for child's age at testing, household assets, male sex, nulliparity, and maternal education.

^7^Adjusted for child's age at testing, maternal education, and male sex.

^8^There were 15 sweet food and beverage items out of a total of 30 food and beverage items. Possible preference score ranges from −15 to +30.

^9^Adjusted for child's age at testing, maternal education, prepregnancy BMI, and distance to weekly market.

In the sensitivity analysis, in which we included a food or beverage item only if the child knew the item, the findings were similar to those from the main analysis: the LNS group was not inferior to the non-LNS group in their preference for sweet items as defined by the number of sweet items chosen during the first level of the photo game (Table 2). The number of sweet items selected among the top 5 items (level 2) was also similar between the 2 groups (Table 2).

Out of a total possible score of 30, the median (IQR) preference score for sweet items was 25 (21, 28) and did not significantly differ between LNS and non-LNS groups (Table 2). We ran a sensitivity analysis in which we treated “don't know” and “never offered/tried” responses as missing values and the results were the same: the preference scores did not differ between LNS and non-LNS groups (*P* > 0.1).

The number of sweet items chosen in the first part of the photo game (when children chose as many items as they wanted out of 30 items) tended to be correlated with caregiver report of preference for sweet snacks (*r* = 0.07, *P* = 0.08), but not for SSB (*r* = −0.01, *P* = 0.8). When we included in the analysis only items that the child recognized, the number of sweet items selected in the photo game (level 1) was correlated with caregiver-reported preference for sweet snacks (*r* = 0.09, *P* = 0.033) and tended to be associated with the caregiver-reported preference for sweet snacks and beverages (*r* = 0.07, *P* = 0.07).

When we included fruits in the list of “sweet food and beverage” items, we observed that the number of sweet items chosen during the photo game was not higher in the LNS group compared to the non-LNS group **(Supplemental Table 4)**. Adjustment for covariates did not alter this finding. In addition, there were no group differences in the preference score for sweet items in unadjusted and adjusted analysis (Supplemental Table 4).

### Children's consumption of sweet foods and beverages

Based on caregiver surveys, 99% of children in our sample had reportedly consumed a sweet food or SSB at least once in the past week. On average, children had consumed sweet foods and SSB [median (IQR)] 15 (9, 22) times in the past week [**Table 3**, LNS, 14 (8, 23); non-LNS, 16 (9, 22)]. About 93% of children had consumed SSB at least once in the past week. On average, children had consumed SSB 5 (3, 10) times in the past week [Table 3, LNS, 5 (3, 10); non-LNS, 6 (2.5, 9)], with over 80% consuming them at least 3 times in the past week. Sweet pastries were the most commonly consumed sweet snack item and were reported to have been consumed 5 (2, 9) times in the past week.

**TABLE 3:** Caregiver report of consumption of sweet foods, sugar-sweetened beverages, and peanut-containing foods in the past week among 4–6-y-old Ghanaian children who participated in the iLiNS-DYAD-G2 follow-up study, by intervention group^1^

		Non-LNS Group (*n* = 640)Median (Q1, Q3)	Adjusted for child age at follow-up		Adjusted for baseline and other covariates	
			LNS vs. Non-LNS		LNS vs. Non-LNS	
Variable	LNS Group (*n* = 345) Median (Q1, Q3)		Difference in means (95% CI)	*P* value	Difference in means (95% CI)	*P* value
Number of times child consumed sweet foods and beverages in the past week^2^	14 (8, 23)	16 (9, 22)	0.06 (−1.29, 1.41)	0.9	−0.23 (−1.58, 1.12)^3^	0.7
Number of times child consumed sugar-sweetened beverages in the past week^4^	5 (3, 10)	6 (2.5, 9)	0.12 (−0.63, 0.86)	0.7	−0.01 (−0.75, 0.73)^5^	0.9
Number of times child consumed peanut-containing foods in the last 7d	2 (0, 3)	2 (1, 4)	−0.11 (−0.44, 0.22)	0.5	−0.14 (−0.47, 0.19)^6^	0.4

^1^Group differences were compared using multiple linear regression and ANCOVA models. LNS, lipid-based nutrient supplement; non-LNS, no exposure to lipid-based nutrient supplement (control group); Q, quartile.

^2^Group differences were compared using a noninferiority margin of 1.96. Noninferiority testing should take precedence over the *P* value (see Figure 2B for graph).

^3^Adjusted for prepregnancy BMI, maternal age, maternal education, nulliparity, and household assets.

^4^Group differences were compared using a noninferiority margin of 1.08. Noninferiority testing should take precedence over the *P* value (see Figure 2C for graph).

^5^Adjusted for prepregnancy BMI, maternal education, nulliparity, and household assets.

^6^Adjusted for maternal education and distance to weekly market.

The mean unadjusted difference (95% CI) between the LNS and the non-LNS groups in the frequency of consumption of sweet foods and beverages and in the frequency of consumption of SSB was 0.06 (−1.29, 1.41) and 0.12 (−0.63, 0.86), respectively. The upper level of the 95% CI of these mean differences between the groups (unadjusted or adjusted for covariates) did not exceed the noninferiority margins (Figure 2B, Figure 2C), indicating that the consumption of these items was not higher in the LNS group compared to the non-LNS group. When we included fruits in the list of “sweet food and beverage” items, we observed that the frequency of consumption of these items was not higher in the LNS group compared to the non-LNS group (Supplemental Table 4). Adjusting for covariates did not alter this finding (Supplemental Table 4).

### Children's consumption of other foods and beverages

Three out of every 4 children had consumed peanut-containing foods in the past week. On average, children had consumed a peanut-containing food or snack [median (IQR)] 2 (1, 4) times in the past week [**Supplemental Table 5**, LNS, 2 (0, 3); non-LNS, 2 (1, 4)]. One-fifth (21.3%) of children had not consumed any fruit in the week preceding the interview. All children had consumed some type of vegetable at least once in the past week, mostly tomatoes and onions, which are commonly included as basic ingredients in soups, stews, and sauces [Supplemental Table 5, 28 (18, 34) times]. Excluding tomatoes and onions, children had consumed other vegetables 4 (2, 7) times in the past week. Animal source foods including meat, fish, and eggs were consumed 11 (7, 17) times in the past week. More fish [6 (3, 14)] was consumed in the past week than meat [2 (0, 3)] or eggs [2 (1, 3)].

Consumption of peanut-containing foods and other foods did not significantly differ between LNS and non-LNS groups (Supplemental Table 5 for all).

## Discussion

In this follow-up study to assess the long-term impact of supplementation with a slightly sweet peanut-based nutrient supplement (LNS) early in life on sweet food and beverage preference and consumption later in childhood, we found that children who were exposed in pre- and postnatal periods to LNS did not have an increased preference for sweet foods and beverages, nor did they consume more sweet items at age 4–6 y compared to children who were never exposed to LNS. Likewise, there were no differences between the groups in the frequency of consumption of peanut-containing foods.

To our knowledge, this is the first examination of the long-term effect of early exposure to a slightly sweet supplement on sweet food preferences using data from a randomized trial. One previous observational study included a follow-up of children to examine the long-term association between feeding sweetened water early in life and the sweet taste preference of children later in childhood. In that study, Beauchamp and colleagues examined the preference for sucrose solution relative to water among a group of US children at birth and at ages 6 mo (16) and 2 y (17). Babies who were routinely fed sweetened water (water sweetened with sugar or honey) during the first months of life compared to babies who were rarely fed sugar water exhibited a greater preference for sweetened water when tested at age 6 mo and later at age 2 y. However, there were no differences between the 2 groups in the preference for a fruit-flavored drink (Kool-Aid) at the age 2-y follow-up (17). It is known that the food matrix in which a taste experience occurs is important and the effects of experience are specific to the particular food context in which sweetness is experienced (35). In the above study (17), children who had never been fed Kool-Aid by their mothers ingested significantly less Kool-Aid, regardless of whether it was sweetened, than children who had previous experience with Kool-Aid. Dietary experience shapes children's development of a sense of what should or should not taste sweet rather than their hedonic response to sweetness in general (35). This could partly explain why we did not observe an increase in the preference for or consumption of sweet foods and beverages in the LNS group compared to the non-LNS group in our study. Our findings are consistent with a recent systematic review that found no evidence to suggest an association between dietary exposure to sweet foods or beverages and the subsequent general acceptance of sweet foods or beverages in the long term among children (20). The studies included in the review were, however, heterogeneous in study design, time and duration of exposure, comparison group, and outcome measured.

In addition, it is possible that in our study, prior exposure to other sweet foods and beverages during the intervention period from ages 6–18 mo was high and did not differ between the groups. This is supported by survey data available from Ghana indicating that ∼30% of children aged 6–23 mo consume sugary foods (36), and by unpublished data from our own study showing that a high proportion of children in the parent trial at age 18 mo consumed sweet foods (50%) and SSB (50%) the previous day. This high level of exposure could explain why we found no group differences. It is also likely that the sugar content of LNS was not high enough to result in a shift in preference for sweet food items. A daily dose (20 g, 118 kcal) of the supplement contains 4 g total sugars, which includes 1.6 g added sugar. To put this in perspective, a serving of a typical SSB (e.g., Coca Cola, 355 mL) providing about 150 kcal, contains ∼39 g of sugar.

We observed that sweet foods and beverages were frequently consumed in this 4–6-y-old population. This is concerning given the potential impact of this intake pattern on health outcomes later in life including weight gain, diabetes, and other chronic diseases (5–7, 37). Understanding a caregiver's motivations for purchasing and feeding sweet foods and beverages to their children will help inform interventions to change the food consumption patterns of these children. In addition, promoting healthier food and beverage choices for children may require regulation of marketing to children (38–41).

We did not observe any significant differences between the 2 groups in the consumption of peanut-containing foods at age 4–6 y. This was a bit surprising because the children were exposed to peanut flavor from LNS prenatally and postnatally and we expected this to have had an influence on their preference for peanut-containing foods, given that flavor preferences can be learned via exposure to flavors in amniotic fluid and breastmilk (42, 43). It is possible that we did not find differences because peanut consumption was common among both groups. About 80% of Ghanaians reportedly consume peanuts at least once per week (44). During the study period when the intervention group received LNS, we did not give any instructions to women in the non-LNS group regarding feeding of peanut-containing foods to their children.

Our study had a number of strengths. We successfully followed up and obtained data from 80% of participants who were part of the parent trial and were eligible for the follow-up study. Both LNS and non-LNS groups remained balanced across most maternal, child, and household characteristics. All data collectors and analysts were blinded to the group assignment of study participants to prevent bias. In our study, caregivers were interviewed at home to obtain information regarding their child's food and beverage consumption, before they brought their child to the test center to undergo the photo game test. This arrangement was helpful in ensuring that caregivers were not biased in their responses based on their observation of their child's response during testing. Lastly, we measured sweet food and beverage preference among children using 2 different methods: an innovative photo game (which measures preference directly) and caregiver-administered interviews. Child preferences ascertained from both methods were correlated, but not strongly. The limitations of each of the methods do not allow for assessing which technique is better for determining preference. In future studies examining similar outcomes, we would recommend collection of additional information by asking the child how much he/she likes each food item listed in the photo game (e.g., by means of “smiley” faces) and then comparing the results to caregiver-reported preferences.

A few limitations deserve mention. First, the innovative photo game method used was not validated in our study population. However, this method was adapted from a similar method used by Boyland and colleagues to measure food preferences among European children (33). To the best of our knowledge, there is no “gold standard” to which we could have compared the photo game. One strategy would have been to compare it to actual intake (although intake is not necessarily a good proxy for subjective preferences). However, assessing actual intake among children of this age is very difficult because caregivers are not necessarily aware of everything the child eats (e.g., at preschool) and the children themselves are too young to self-report quantitative intake information. A second limitation is that information on child's food and beverage consumption was based on maternal recall, hence there is the potential for bias. However, we have no reason to believe that reporting bias was differential between groups. Lastly, we observed differential loss to follow-up between the groups, with a higher loss in the non-LNS group. However, similarities in most maternal, household, and child characteristics between intervention groups and also between participants included in the analysis and those lost to follow-up suggest that bias is not a substantial concern.

We conclude that the provision of a slightly sweet, peanut-containing LNS to mothers during pregnancy and lactation and to children from ages 6–18 mo, compared to no supplementation with LNS, did not increase children's preference for or consumption of sweet foods and beverages, nor their consumption of peanut-containing foods, at age 4–6 y. Research is needed to examine whether this finding holds true in populations with a lower level of exposure to other sweet foods and beverages during infancy and preschool years.

## Supplementary Material

nxy293_Supplement_File

## Notes

Author disclosures: HO, MA, RRY, CDA, SA-A, SMT, HJB, MEO, SMK, BMO, and KGD, no conflicts of interest.

Supplemental Tables 1–5 are available from the “Supplementary data” link in the online posting of the article and from the same link in the online table of contents at https://academic.oup.com/jn/.

Abbreviations used: IFA, iron and folic acid; iLiNS, International Lipid-based Nutrient Supplement; LNS, lipid-based nutrient supplement; MMN, multiple micronutrients; SSB, sugar-sweetened beverages.
