# Supplementary material for: Exposure to a Slightly Sweet Lipid-Based Nutrient Supplement During Early Life Does Not Increase the Preference for or Consumption of Sweet Foods and Beverages by 4–6-y-Old Ghanaian Preschool Children: Follow-up of a Randomized Controlled Trial
Source: J Nutr. 2019 Feb 15;149(3):532–41. doi: 10.1093/jn/nxy293 (PMC6398382; doi:10.1093/jn/nxy293)
Supplement: nxy293_Supplement_File [file nxy293_supplement_file.pdf]

Supplementary data

Supplemental Table 1: Food categories included in the photo game and caregiver report of child food and beverage preference	
Sweet, low nutrient-dense beverages	New star drink<br>Kalyppo<br>Fanta<br>Sobolo (sweetened drink made from hibiscus leaves)<br>Coca-cola
Sweet, low fat, low nutrient-dense items	Sugar-cane<br>Tomtom toffee<br>Agatha toffee<br>Chocomilo<br>Fanyogo
Sweet, high fat, low nutrient-dense items	Doughnut<br>Cake<br>Chocolate<br>Bofrot (fried dough)<br>Atsormor (fried pastry)
Savory, high fat, low nutrient-dense items	Plantain chips<br>Fried plantain<br>Fried sweet potato<br>Fried cassava balls<br>Meat pie
Fruits	Mango<br>Pineapple<br>Water-melon<br>Pawpaw<br>Orange
Vegetables	Cabbage<br>Tomatoes<br>Green pepper<br>Cucumber<br>Carrot

Supplementary data

Supplemental Table 2: Maternal and child characteristics of participants who were included in analysis compared to participants lost to follow-up (for caregiver report of child food preference and consumption)[1]

Variable	Included (n=985)	Lost to follow-up (n=335)	P-value
Maternal characteristics) at time of enrolment into the parent trial			
Age (y)	26.8 ± 5.4	26.8 ± 5.4	0.2
Education (y)	7.6 ± 3.5	7.6 ± 4.0	0.8
Married or cohabiting n (%)	919 (93.3)	305 (91.0)	0.2
Pre-pregnancy BMI (kg/m^2)	24.6 ± 4.5	24.3 ± 4.1	0. 3
Nulliparity n (%)	315 (32.0)	131 (39.1)	0.017
Household assets score (#)[3]	0.03 ± 0.96	-0.06 ± 1.10	0.2
Distance to market (m)	1239 (655, 2327)	1157 (523, 2258)	0.2
Child characteristics			
Male sex n (%)	474 (48.2)	137 (52.1)	0.2

[1]Values represent mean ± SD or n (%) or median (q1, q3). Group differences were compared using ANOVA for continuous variables and the chi-squared test for categorical variables.

[2]Proxy indicator for socio-economic status constructed for each household based on ownership of a set of assets (radio, television, refrigerator, cell phone, and stove), lighting source, drinking water supply, sanitation facilities, and flooring materials. Household ownership of these assets was combined into an index (with a mean of zero and standard deviation of one) using principal components analysis. Higher values represent higher socioeconomic status.

Supplementary data

Supplemental Table 3: Child, maternal and household characteristics for participants who were included in analysis compared to participants lost to follow-up (for the photo game)[1]

**Variable**	**Tested at follow-up N=624**	**Loss to follow-up N=151**	**P-value**
Maternal characteristics at time of enrolment into the parent trial			
Age (y)	26.9 ± 5.6	25.2 ± 4.9	0.001
Education (y)	7.7 ± 3.6	7.9 ± 3.8	0.5
Married or cohabiting % (n)	92.7 (579)	85.4 (129)	0.004
Maternal pre-pregnancy BMI (kg/m^2)	24.7 ± 4.4	23.7 ± 3.9	0.015
Nulliparity % (n)	32.7 (204)	45.7 (69)	0.003
Household speaks Krobo as main language % (n)	72.9 (455)	68.2 (103)	0.2
Household assets score[2] (#)	0.01 ± 0.97	-0.05 ± 1.04	0.5
Household Food Insecurity Access Scale (#)	2.5 ± 4.0	2.9 ± 4.4	0.3
Distance to market (m)	1235 (670, 2356)	1056 (518, 2147)	0.025
Child characteristics			
Male sex % (n/N)	48.7 (304/624)	50.0 (65/130)	0.8

[1]Values represent mean ± SD or n/N (%) or median (q1, q3); Group differences were compared using ANOVA for continuous variables and the chi-squared test for categorical variables.

[2]Proxy indicator for household socioeconomic status constructed for each household based on ownership of a set of assets (radio, television, refrigerator, cell phone, and stove), lighting source, drinking water supply, sanitation facilities, and flooring materials. Household ownership of these assets was combined into an index (with a mean of zero and standard deviation of one) using principal components analysis. Higher values represent higher socioeconomic status.

Supplementary data

Supplemental Table 4: Food and beverage consumption (# times consumed per week) among 4 to 6-year old Ghanaian children who participated in the iLiNS DYAD-G2 follow up study[1]

Variable	All groups combined (n=985)	LNS Group (n=345)	Non-LNS Group (n=640)	P-value
	Median (IQR)	Median (IQR)	Median (IQR)	
Sweet foods and beverages	15 (9, 22)	14 (8, 23)	16 (9, 22)	0.9
Sugar sweetened beverages	5 (3, 10)	5 (3, 10)	6 (2.5, 9)	0.8
Sweet snacks/foods	8 (4, 13)	8 (4, 13)	9 (4, 14)	0.9
Peanut-containing foods	2 (1, 4)	2 (0, 3)	2 (1, 4)	0.5
Savory snacks	2 (1, 4)	2 (0, 4)	2 (1, 4)	0.8
Milk	0 (0, 1)	0 (0, 1)	0 (0, 1)	0.9
Fruits	3 (1, 6)	3 (1, 6)	3 (1, 6)	0.6
Vegetables[2]	28 (18, 34)	28 (18, 34)	28 (18, 34)	0.9
Vegetables[3]	4 (2, 7)	3 (2, 7)	4 (2, 7)	0.2
Legumes, nuts and seeds	5 (3, 8)	5 (3, 8)	5 (3, 8)	0.3
Animal-source foods[4]	11 (7, 17)	11 (7, 17)	11 (7, 17)	0.9
Meat	2 (0, 3)	1 (0, 3)	2 (0, 3)	0.4
Fish	6 (3, 14)	7 (3, 14)	6 (3, 13)	0.5
Eggs	2 (1, 3)	2 (1, 3)	2 (1, 3)	0.1

[1]Data presented as number of times a particular food/beverage item was consumed in the week preceding the interview; Values represent median (IQR); LNS = lipid-based nutrient supplement; Non-LNS = no exposure to LNS (control group); Group differences were examined using negative binomial modelling techniques.

[2]Includes tomatoes and onions.

[3]Excludes tomatoes and onions.

[4]Includes meat, fish, eggs and “wele” (cow hide).

Supplementary data

Supplemental Table 5: Sweet food (including fruits) and sugar-sweetened beverage preference among 4 to 6-year old Ghanaian children who participated in the iLiNS DYAD-G2 follow up study, by intervention group[1]

Variable	LNS Group	Non-LNS Group	Adjusted for child age at follow-up		Adjusted for baseline and other covariates	
			LNS vs. No LNS		LNS vs. No LNS	
	Median (Q1, Q3)	Median (Q1, Q3)	Difference in means (95% CI)	P-value	Difference in means (95% CI)	P-value
**Preference as assessed in photo game (*n*=624)**						
All food/beverage items						
Total items chosen out of 30 items	28 (19, 30)	28 (21, 30)				
Number of sweet items (including fruits) chosen by child from among 30 items[2]	15 (11, 15)	15 (11, 15)	0.00 (-0.70, 0.70)	0.9	0.03 (-0.67, 0.72)[3]	0.9
Number of sweet items (including fruits) chosen by child (out of top 5 favorite food/ beverage items)	3 (3, 4)	3 (2, 4)	-0.01 (-0.21, 0.18)	0.9	-0.01 (-0.21, 0.18)[4]	0.9
Only food/ beverage items that were known[5]						
Total items chosen	14 (10, 18)	14 (10, 17)				
Number of sweet items (including fruits) chosen by child from among 30 items[2]	8 (5, 10)	8 (5, 9)	0.04 (-0.54, 0.63)	0.9	0.15 (-0.43, 0.74)[6]	0.6

Supplementary data

Number of sweet items (including fruits) chosen by child (out of top 5 favorite food/ beverage items)	2 (1, 3)	2 (1, 3)	0.04 (-0.19, 0.27)	0.7	0.06 (-0.17, 0.29)[7]	0.6
**Preference as reported by caregiver (*n*=985)**						
Preference score for sweet food/ beverage items (including fruits) (as reported by caregiver)[8]	25 (21, 28)	25 (21, 28)	0.11 (-0.80, 1.01)	0.8	0.01 (-0.88, 0.91)[9]	0.9
**Consumption as reported by caregiver (*n*=985)**						
Number of times child consumed sweet foods (including fruits) and sugar-sweetened beverages in the past week[10]	14 (8, 23)	16 (9, 22)	0.06 (-1.29, 1.41)	0.9	-0.23 (-1.58, 1.12)[11]	0.7

[1]Group differences compared using multiple linear regression and ANCOVA models; LNS = lipid-based nutrient supplement; Non-LNS = no exposure to LNS (control group).
[2]Group differences were compared using a non-inferiority margin of 0.88 for this outcome (based on SD of 4.4 when fruits were included in the list of sweet items; equivalent to an effect size of 0.2). Non-inferiority testing should take precedence over the p-value.
[3]Adjusted for distance to weekly market.
[4]Adjusted for child age only.
[5]This sub-analysis only included food/ beverage items that were known and recognized by the child (based on specific questions described in methods section).
[6]Adjusted for child's age at testing, household assets and maternal education.
[7]Adjusted for child's age at testing and maternal education.
[8]There were 20 sweet food and beverage items out of 30 food and beverage items. Possible preference score ranges from -20 to +40.
[9]Adjusted for distance to weekly market.
[10]Group differences were compared using a non-inferiority margin of 2.28 for this outcome (based on SD of 12.2 when fruits were included in the list of sweet items; equivalent to an effect size of 0.2). Non-inferiority testing should take precedence over the p-value.
[11]Adjusted for pre-pregnancy BMI, woman's age, education, nulliparity and household assets.
